# Apple-Derived Pectin Modulates Gut Microbiota, Improves Gut Barrier Function, and Attenuates Metabolic Endotoxemia in Rats with Diet-Induced Obesity

**DOI:** 10.3390/nu8030126

**Published:** 2016-02-29

**Authors:** Tingting Jiang, Xuejin Gao, Chao Wu, Feng Tian, Qiucheng Lei, Jingcheng Bi, Bingxian Xie, Hong Yu Wang, Shuai Chen, Xinying Wang

**Affiliations:** 1 Department of General Surgery, Jinling Hospital, Medical School of Nanjing University, Nanjing 210002, China; jiangtingting08med@163.com (T.J.); xuejingao870214@163.com (X.G.); wuchao0008@163.com (C.W.); tianfeng_nju@163.com (F.T.); lqiuchenggd@163.com (Q.L.); ahbijingcheng@163.com (J.B.); 2 Department of General Surgery, South Medical University, Guangzhou 510515, China; 3 State Key Laboratory of Pharmaceutical Biotechnology and MOE Key Laboratory of Model Animal for Disease Study, Model Animal Research Center, Nanjing University, Pukou District, Nanjing 210061, China; xiebx@nicemice.cn (B.X.); wanghy@nicemice.cn (H.Y.W.)

**Keywords:** obesity, apple-derived pectin, gut microbiota, gut barrier function, metabolic endotoxemia

## Abstract

This study was aimed at determining potential effects of apple-derived pectin on weight gain, gut microbiota, gut barrier and metabolic endotoxemia in rat models of diet-induced obesity. The rats received a standard diet (control; Chow group; *n* = 8) or a high-fat diet (HFD; *n* = 32) for eight weeks to induce obesity. The top 50th percentile of weight-gainers were selected as diet induced obese rats. Thereafter, the Chow group continued on chow, and the diet induced obese rats were randomly divided into two groups and received HFD (HF group; *n* = 8) or pectin-supplemented HFD (HF-P group; *n* = 8) for six weeks. Compared to the HF group, the HF-P group showed attenuated weight gain (207.38 ± 7.96 g *vs.* 283.63 ± 10.17 g, *p* < 0.01) and serum total cholesterol level (1.46 ± 0.13 mmol/L *vs.* 2.06 ± 0.26 mmol/L, *p* < 0.01). Compared to the Chow group, the HF group showed a decrease in Bacteroidetes phylum and an increase in Firmicutes phylum, as well as subordinate categories (*p* < 0.01). These changes were restored to the normal levels in the HF-P group. Furthermore, compared to the HF group, the HF-P group displayed improved intestinal alkaline phosphatase (0.57 ± 0.20 *vs.* 0.30 ± 0.19, *p* < 0.05) and claudin 1 (0.76 ± 0.14 *vs.* 0.55 ± 0.18, *p* < 0.05) expression, and decreased Toll-like receptor 4 expression in ileal tissue (0.76 ± 0.58 *vs.* 2.04 ± 0.89, *p* < 0.01). The HF-P group also showed decreased inflammation (TNFα: 316.13 ± 7.62 EU/mL *vs.* 355.59 ± 8.10 EU/mL, *p* < 0.01; IL-6: 51.78 ± 2.35 EU/mL *vs.* 58.98 ± 2.59 EU/mL, *p* < 0.01) and metabolic endotoxemia (2.83 ± 0.42 EU/mL *vs.* 0.68 ± 0.14 EU/mL, *p* < 0.01). These results suggest that apple-derived pectin could modulate gut microbiota, attenuate metabolic endotoxemia and inflammation, and consequently suppress weight gain and fat accumulation in diet induced obese rats.

## 1. Introduction

In recent years, obesity and related metabolic disorders have emerged as major health concerns [1,2]. Obesity is associated with increased risks for developing type 2 diabetes mellitus (T2DM), hyperlipidemia, hypertension, coronary heart disease (CHD), stroke, and cancer. Development of these diseases can also lead to psychological and psychiatric illnesses, adding to the societal burden associated with these diseases [3,4,5].

Obesity and related metabolic disorders are attributable to a combination of genetics, unhealthy diet and lifestyle. Recent studies have demonstrated that disturbance of gut microbiota, especially the ratio of Bacteriodetes to Firmicutes phylum, is closely related to obesity and metabolic disorders [6,7,8,9,10,11]. In addition, obese subjects exhibit systemic chronic inflammation and a high level of serum endotoxins (lipopolysaccharides (LPS), a key component of the cell wall of gram-negative bacteria), termed “metabolic endotoxemia”, which associates with gut barrier dysfunction [12,13,14,15,16].

Gut microbiota in the lumen is normally isolated by the intestinal epithelium from lamina propria and deeper layers [17,18], and LPS derived from gut microbiota is confined to the gut lumen and does not penetrate healthy intestinal epithelium [19]. However, a damaged intestinal epithelium or other gut barrier dysfunction can lead to disturbance of gut microbiota [16], allow for LPS permeation and cause metabolic endotoxemia [20,21,22]. Recognition of LPS by Toll-like receptor 4 (TLR4) of host cells triggers downstream inflammatory events [23,24] that contributes to the development of obesity and metabolic disorders such as insulin resistance [10,25].

Tight junctions are key components for maintaining gut barrier integrity [17,26]. Another important protein for gut barrier function is intestinal alkaline phosphatase (IAP) that is a type of glycoprotein anchored in the apical membrane of enterocytes. IAP has multiple roles in maintenance of gut barrier, including detoxification of LPS via its dephosphorylation, remission of systemic inflammation, protection of gut barrier function and modulation of gut microbiota [27,28].

Although various approaches are recommended for obesity management [29], such as dieting, behavior therapy, exercise, pharmacotherapy, and bariatric surgery, they are often defective [29,30]. Here, we consider that modification of gut microbiota, protection of gut barrier, remission of metabolic endotoxemia, and relief of systemic inflammation may provide a novel strategy for the treatment of obesity and related metabolic disorders.

Dietary fiber consists of non-digestible carbohydrates that derived from plants. Recent animal experiments and clinical trials have shown that dietary fiber, such as whole-grain cereal, grape skin extract, yellow pea fiber and wheat-derived arabinoxylan oligosaccharides, has hypolipidemic and hypoglycemic effects and may contribute to weight loss [31,32,33,34,35]. Apple-derived pectin is the main soluble fiber in apples and can be fermented by gut microbiota in the colon to produce metabolites with local intestinal and systemic effects. Apple-derived pectin may also help to maintain the balance of gut microbiota [36]. 

The aim of the present study was to assess potential effects of apple-derived pectin on diet-induced obesity in rats. We found that apple-derived pectin could modulate gut microbiota, preserve gut barrier function, and alleviate metabolic endotoxemia and inflammation in diet-induced obese rats. Our findings suggest that apple-derived pectin may be useful for the clinical management of obesity.

## 2. Materials and Methods

### 2.1. Animals

Male Sprague-Dawley rats were obtained from the Medical Experiment Animal Center of the Jinling Hospital, Nanjing, China, at 4 weeks of age with an initial weight of 90 ± 10 g. Rats were housed in individual cages in an optimum environment at 23 ± 2 °C and a relative air humidity of 55% ± 10% with a 12 h light/dark cycle. Animals had free access to a standard chow diet (10% kcal% fat; D12450J, Research Diets, New Brunswick, NJ, USA) or a high fat diet (60% kcal% fat; D12492, Research Diets) and water throughout the experiment. This study was approved by the Animal Care and Use Committee of Nanjing University and Jinling Hospital and complied with the principles of laboratory animal care (NIH publication No. 86–23, revised 1985).

### 2.2. Diet and Study Design

Rats were randomized into two groups and received either a standard chow diet (Chow group, *n* = 8) as a control or a HFD (*n* = 32) to induce obesity for 8 weeks. Body weight and food intake were recorded every week. 

After high fat feeding, diet induced obese rats were selected as previously described [37], wherein the top 50th percentile of weight gainers were randomized for the following interventions. Sixteen were randomized to receive either a HFD (HF group, *n* = 8) or a HFD supplemented with pectin (5% wt/wt) (HF-P group, *n* = 8) for 6 weeks. The Chow group continued a standard chow diet for 6 weeks. Body weight and food intake were recorded every week. 

### 2.3. Sample Collection

All animals were anesthetized by intraperitoneal administration of ketamine (0.3 mL/100 g body weight). Blood (about 3 mL) was immediately collected in a dry tube without heat source. Blood samples were allowed to clot for 2 h at room temperature and were then centrifuged for 15 min at 3000 rpm at 4 °C. Serum was removed, and the samples were stored at −80 °C until further analysis.

After blood collection, a part of the liver, distal ileum, mesentery adipose, and the whole part of epididymal fat pad were collected, weighed, wrapped, and immediately put into liquid nitrogen. This process took no longer than 3 min after sacrifice of the animal. All samples were stored frozen at −80 °C until further analysis.

### 2.4. Body Weight and Adipose Tissue Wet Weight

Body weight and adipose tissue wet weight were measured using an electronic scale. Body weight was measured once a week and adipose tissue wet weight was measured after the rats were sacrificed.

### 2.5. Blood Parameters

Serum glucose, triglycerides, and total cholesterol concentrations were measured by enzyme linked immunosorbent assay (ELISA) kits in accordance with the manufacturer’s instructions (Labassay™ Wako kit, Wako Pure Chemical Industries, Ltd., Osaka, Japan). Serum insulin concentrations were measured using an ELISA kit (Millipore Corp., Billerica, MA, USA).

### 2.6. Measurement of Serum LPS

Serum LPS concentration was determined by Chromogenic End-point Tachypleus Amebocyte Lysate (CE TAL) assay (Chinese Horseshoe Crab Reagent Manufactory, Co., Ltd., Xiamen, China). In this assay, color intensity is directly proportional to endotoxin levels. Serum was diluted 1/10 with pyrogen-free pipes to avoid interference in the reaction. Endotoxin in the serum activates a cascade of enzymes in the assay, and the activated enzyme splits the synthetic substrate, producing a yellow product with maximum absorbance at 405 nm. The yellow product can further react with diazo reagents to form a purple product with maximum absorbance at 545 nm. Every sample was treated in duplicate for determination. The limit of detection ranged from 0.1 to 1.0 EU/mL. An internal control for LPS recovery was included in the calculation. Every reaction in the kit was done in duplicate.

### 2.7. Western Blot

Proteins of ileum or liver samples were separated by sodium dodecyl sulfate (SDS)-polyacrylamide gel electrophoresis and transferred to polyvinylidene fluoride (PVDF) membranes. After blocking with skim milk (BioRad, Hercules, CA, USA), membranes were incubated at 4 °C overnight using the antibodies indicated. Band density was detected by horseradish-peroxidase conjugated secondary antibodies (Promega, Madison, WI, USA) and ECL (enhanced chemiluminescence reagent; GE Healthcare, Chalfont St. Giles, UK). Bands located in a predicted molecular weight were used to verify targeted proteins. β-actin or glyceraldehyde-3-phosphate dehydrogenase (GAPDH) was used as an internal control to adjust the density of bands on multiple membranes. For quantification of signals, the images were quantified by an Image J software (Wayne Rasband, National Institutes of Health, Bethesda, MD, USA).

### 2.8. Quantitative RT-PCR Analysis

mRNA levels of TNF α, IL-6, IL-10, and TLR4 were measured by quantitative real-time polymerase chain reaction (qPCR). The primers are listed in Table 1.

mRNA extraction and RT-PCR were performed according to the manufacturer’s instructions as described in the PrimeScript RT reagent Kit (TaKaRa Bio, Tokyo, Japan). Q RT-PCR was performed by SYBR Select Master Mix System (Applied Biosystems, Foster City, CA, USA). The levels of mRNA expression were measured by StepOne Realtime PCR system with a ∆Ct relative quantification model. The mRNA expression of βactin and 36B4, two reference genes, were calculated and used for normalization. In our study, mRNA expression of the tested genes displayed similar results with either reference gene, thus we used βactin as the reference gene for normalization in this manuscript.

### 2.9. Hematoxylin and Eosin (H & E) Staining

The ileal tissues were processed (Tissue-Tek VIP; Sakura Finetek, Tokyo, Japan), embedded in paraffin wax, and cut into 5-µm thick slices. Paraffin embedding, slicing, and H & E staining were performed according to the standard procedure.

### 2.10. Immunohistochemistry (IHC) Staining

IHC staining was used to detect the location of IAP. After antigen retrieval with buffered citrate and blocking with 5% bovine serum albumin (BSA), ileum tissues were incubated with primary antibodies against IAP (Abcam, Cambridge, UK, 1:200) overnight at 4 °C. The sections were then processed using the DAB Kit (ZSGB-Bio, Beijing, China) according to the manufacturer’s instructions. Hematoxylin staining was performed at the end to counterstain nuclei. The cover slips were fixed with 50% glycerin.

### 2.11. 16S rRNA Pyrosequencing

#### 2.11.1. Collection and Transportation of Samples

Cecum content was collected from every rat, stored in liquid nitrogen, transported to BGI laboratory (Shenzhen, China), packed with dry ice, and then immediately stored in a −80 °C refrigerator before extraction of total DNA.

#### 2.11.2. Detection of Samples

Detection of samples included concentration and sample integrity. Concentration was detected by a fluorometer or microplate Reader, while sample integrity was detected by agarose gel electrophoresis (concentration of agarose gel: 1%; voltage: 150 V; electrophoresis Time: 40 min).

#### 2.11.3. Library Construction

Total DNA was normalized to 30 ng per reaction, and then V4 Dual-index Fusion PCR Primer Cocktail and PCR Master Mix were added to run PCR (melting temperature: 56 °C, PCR cycle: 30). Subsequently, AmpureXP beads (AGENCOURT) were added to the PCR products to remove unspecific products.

#### 2.11.4. Library Validation

The final library was quantitated by real-time quantitative PCR (EvaGreen™, Hayward, CA, USA).

#### 2.11.5. Library Sequencing

Library sequencing was conducted by pair end on MiSeq System, with sequencing strategy PE250 (PE251 + 8 + 8 + 251) (MiSeq Reagent Kit, Illumina Hong Kong Limited, Hong Kong, China). Mothur pipeline and QIIME pipeline were used to analyze the data. 

### 2.12. Statistical Analysis

Data are presented as the mean ± standard deviation (SD), and significant difference among groups was analyzed by one-way analysis of variance (ANOVA) followed by Dunnett’s *post hoc* test (SPSS 21.0, IBM, New York, NY, USA). Significant difference of the body weight among groups was analyzed by repeated measures analysis of variance (SPSS 21.0). Statistical significance was set at *p* < 0.05.

## 3. Results

### 3.1. Apple-Derived Pectin Protected Rats from High Fat Diet Induced Obesity

**Figure 1:**
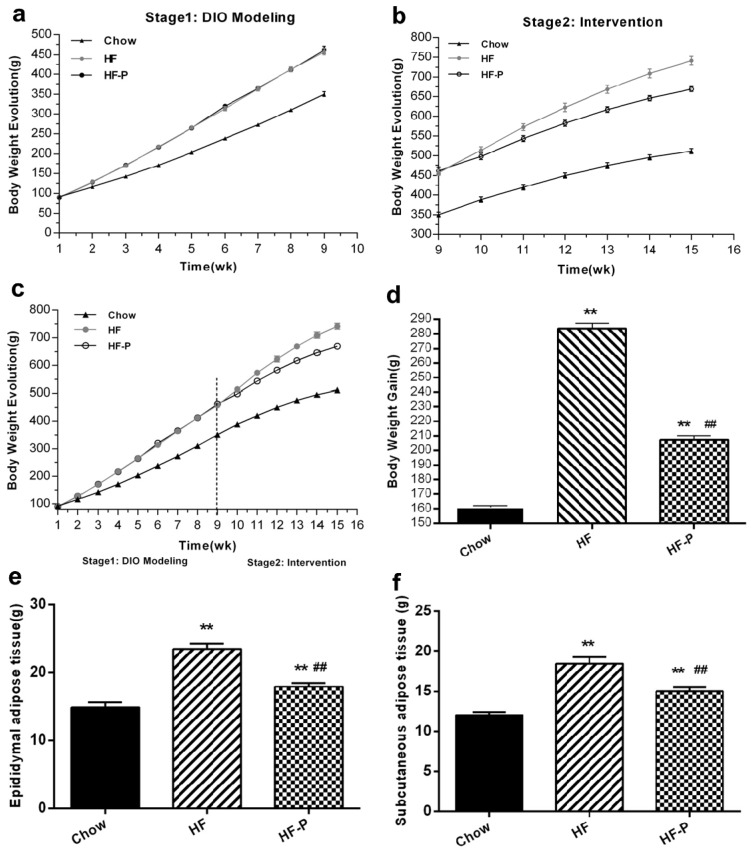
Apple-derived pectin suppresses body weight gain and development of adipose tissue in rats fed a high fat diet. (**a**–**c**) Growth curves; (**d**) body weight gain; (**e**) weight of epididymal adipose tissue; and (**f**) weight of subcutaneous adipose tissue of rats in Chow, high fat diet (HF), and high fat diet supplemented with pectin (HF-P) groups (** *p* < 0.01 *vs.* Chow, ## *p* < 0.01 *vs.* HF, one way analysis of variance (ANOVA)).

In the intervention stage, rats in the high fat diet (HF) group gained more body weight than rats in the Chow group (283.63 ± 10.17 g *vs.* 161.00 ± 2.88 g, *p* < 0.01). Importantly, rats in the HF supplemented with pectin (HF-P) group gained significantly less weight than rats in the HF group (207.38 ± 7.96 g *vs.* 283.63 ± 10.17 g, *p* < 0.01) (Figure 1). Rats in the HF group developed adipose tissue more rapidly than the Chow group (epididymal, 23.44 ± 2.36 g *vs.* 14.86 ± 2.04 g, *p* < 0.01; subcutaneous, 18.44 ± 2.36 g *vs.* 11.99 ± 1.21 g, *p* < 0.01) (Figure 1). Pectin supplementation significantly suppressed the development of adipose tissue in the HF-P group as compared with the HF group (epididymal, 17.90 ± 1.55 g *vs.* 23.44 ± 2.36 g, *p* < 0.01; subcutaneous, 15.02 ± 1.44 g *vs.* 18.44 ± 2.36 g, *p* < 0.01) (Figure 1).

### 3.2. Apple-Derived Pectin Alleviated High Fat Diet Induced Hypercholesterolemia

**Figure 2:**
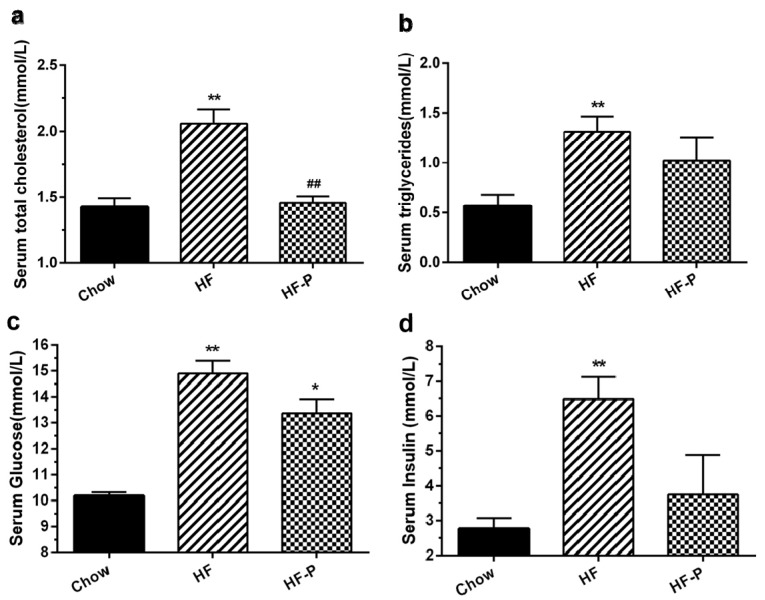
Effects of apple-derived pectin on HFD-induced changes in blood chemistry: (**a**) serum total cholesterol; (**b**) triglycerides; (**c**) glucose; and (**d**) insulin in Chow, HF, and HF-P groups. (** *p* < 0.01 *vs.* Chow, * *p* < 0.05 *vs.* Chow, ## *p* < 0.01 *vs.* HF, one way ANOVA).

Rats in the HF group exhibited higher levels of serum total cholesterol, triglycerides, glucose, and insulin than those in the Chow group (total cholesterol, 2.06 ± 0.26 mmol/L *vs.* 1.43 ± 0.16 mmol/L, *p* < 0.01; triglycerides, 1.31 ± 0.41 mmol/L *vs.* 0.57 ± 0.294 mmol/L, *p* < 0.01; glucose, 15.14 ± 3.85 mmol/L *vs.* 10.18 ± 0.35 mmol/L, *p* < 0.01; insulin, 6.49 ± 1.82 mmol/L *vs.* 3.07 ± 1.14 mmol/L, *p* < 0.01). Total cholesterol levels were significantly decreased in rats of the HF-P group as compared with those in the HF group (1.46 ± 0.13 mmol/L, *p* < 0.01), while serum triglycerides, fasting serum glucose, and insulin levels showed only a trend of decrease within the duration of the study (triglycerides, 1.02 ± 0.65 mmol/L *vs.* 1.31 ± 0.41 mmol/L, *p* = 0.669; glucose, 13.37 ± 1.32 mmol/L *vs.* 15.14 ± 3.85 mmol/L, *p* = 0.607; insulin, 3.75± 3.00 mmol/L *vs.* 6.49 ± 1.82 mmol/L, *p* = 0.167) (Figure 2).

### 3.3. Apple-Derived Pectin Prevented HFD-Induced Alterations of Gut Microbiota

**Figure 3:**
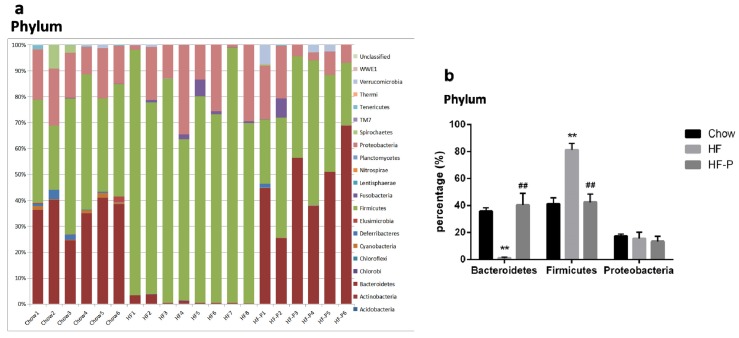

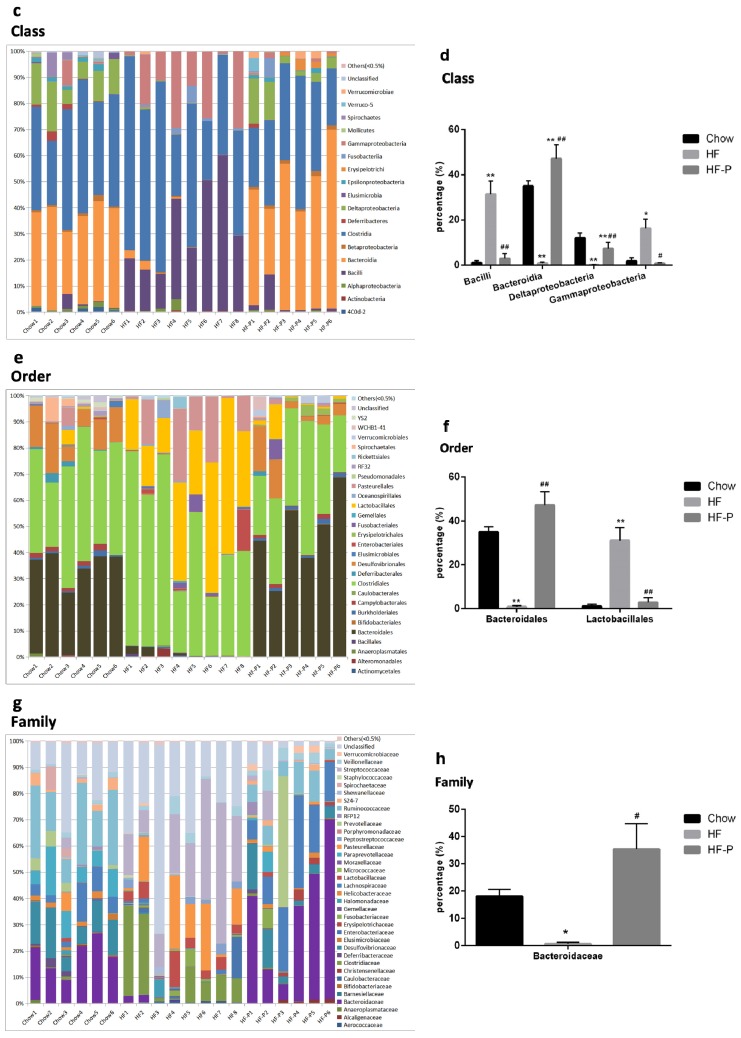

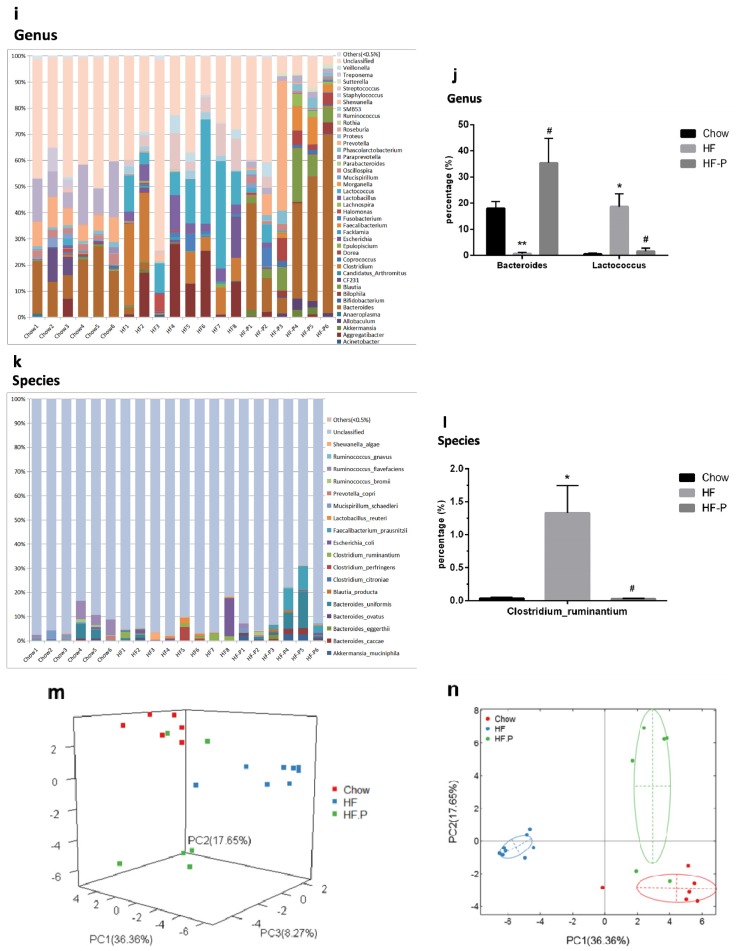
Composition analysis of gut microbiota at the: (**a**,**b**) phylum; (**c**,**d**) class; (**e**,**f**) order; (**g**,**h**) family; (**i**,**j**) genus; and (**k**,**l**) species level in Chow, HF, and HF-P groups; (**m**) Principal component analysis (PCA) and (**n**) clustering analysis of gut microbiota in Chow, HF, and HF-P groups (** *p* < 0.01 *vs.* Chow, * *p* < 0.05 *vs.* Chow, ## *p* < 0.01 *vs.* HF, # *p* < 0.05 *vs.* HF, one way ANOVA).

Gut microbiota was analyzed by 16S rDNA pyrosequencing at the levels of phylum, class, order, family, genus and species (Figure 3). The number of reads per sample and raw data can be found in Supplementary Materials (Table S1).

At the phylum level, rats in the HF group had a significantly lower level of Bacteroidetes and a higher level of Firmicutes than the Chow group. However, in the HF-P groups, these changes were restored to similar levels as the Chow group. There was no significant difference in the level of Proteobacteria among the three groups. 

At the class level, rats in the HF group presented a significantly higher level of Bacilli (a class of Firmicutes phylum) and Gammaproteobacteria (a class of Proteobacteria phylum) as well as a significantly lower level of Bacteroidia (a class of Bacteroidetes phylum) and Deltaproteobacteria (a class of Proteobacteria phylum). The percentage levels of the above four classes of bacteria were reverted to normal in the HF-P group (Chow group as reference).

At the order level, we observed a significantly lower level of Bacteroidales (an order of Bacteroidia class, Bacteroidetes phylum) and a higher level of Lactobacillales (an order of Bacilli class, Firmicutes phylum) in the HF group than in the Chow group. After pectin supplementation for six weeks, the levels of the above two orders of bacteria in the HF-P group were similar to those in the Chow group.

At the family level, we observed a significantly lower level of Bacteroidaceae (a family of Bacteroidales order, Bacteroidia class, Bacteroidetes phylum) in the HF group than in the Chow group, and these decreases were restored to normal levels in the HF-P group.

At the genus level, we observed a dramatically lower level of *Bacteroides* (a genus of Bacteroidaceae family, Bacteroidales order, Bacteroidia class, Bacteroidetes phylum) and a higher level of *Lactococcus* (a genus of Streptococcaceae Family, Lactobacillales Order, Bacilli Class, Firmicutes Phylum) in the HF group than in the Chow group. Again, pectin supplementation prevented these changes induced by high fat diet.

At the species level, *Clostridium ruminantium* (a species of *Clostridium* Genus, Clostridiaceae Family, Clostridiales Order, Clostridia Class, Firmicutes Phylum) was the only species that displayed a significant increase upon high fat diet and became normal with pectin supplementation.

Accordingly, principal component analysis (PCA, Figure 3m) and clustering analysis (Figure 3n) illustrated both similarity and variance among the Chow, HF, and HF-P groups, where the first three components explained 62.28% of the total variance (36.36%, 17.65%, and 8.27% for PC1, PC2, and PC3, respectively). The score plot showed that diversity of gut microbiota was similar between the Chow and HF-P groups but different from the HF group.

Taken together, these data indicated that rats in the HF group exhibited a lower level of Bacteroidetes phylum and a higher level of Firmicutes phylum than those in the Chow group. Importantly, both of these changes were restored to normal levels in the HF-P group. At the downstream level, we found some changes consistent with the phylum level, such as higher levels in the HF group of the Bacilli class, Lactobacillales order, *Lactococcus* genus, *Clostridium ruminantium* species (belonging to the Firmicutes phylum), and a significantly lower level of Bacteroidia class, Bacteroidales order, Bacteroidaceae family, and *Bacteroides* genus (belonging to Bacteroidetes phylum). Supplementation of apple-derived pectin brought these changes back to normal levels (Chow group as reference). 

### 3.4. Apple-Derived Pectin Restored the Expression of Intestinal Alkaline Phosphatase (IAP) in the Ileal Tissueof Rats on High Fat Diet

**Figure 4:**
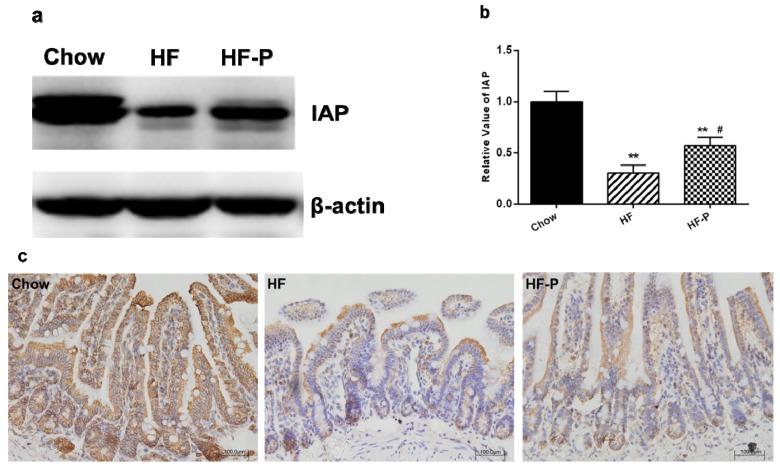
Expression of intestinal alkaline phosphatase (IAP) in the ileum of rats: (**a**) representative immunoblots for IAP and β-actin; (**b**) quantitation of IAP in the Chow, HF, and HF-P groups (** *p* < 0.01 *vs.* Chow, # *p* < 0.05 *vs.* HF, one way ANOVA); and (**c**) immunological histological chemistry analysis of IAP in Chow, HF, and HF-P groups. Original magnification: 20×.

We measured the protein levels of IAP via immunoblotting and found that the expression of IAP in the ileal tissue of rats in the HF group was significantly lower than that in the Chow group (0.30 ± 0.19 *vs.* 1.00 ± 0.25, *p* < 0.01). Supplementation of pectin significantly increased the level of IAP in the ileal tissue of rats as compared with high fat diet alone (0.57 ± 0.20 *vs.* 0.30 ± 0.19, *p* < 0.05) (Figure 4).

Consist with the immunoblotting analysis of IAP, IHC analysis of IAP in the ileum also revealed that high fat diet reduced the expression of IAP in comparison with chow diet while supplementation of pectin could attenuate this reduction.

### 3.5. Apple-Derived Pectin Prevented the High Fat Diet Induced mRNA Expression of TLR4 in the Ileal Issue

**Figure 5:**
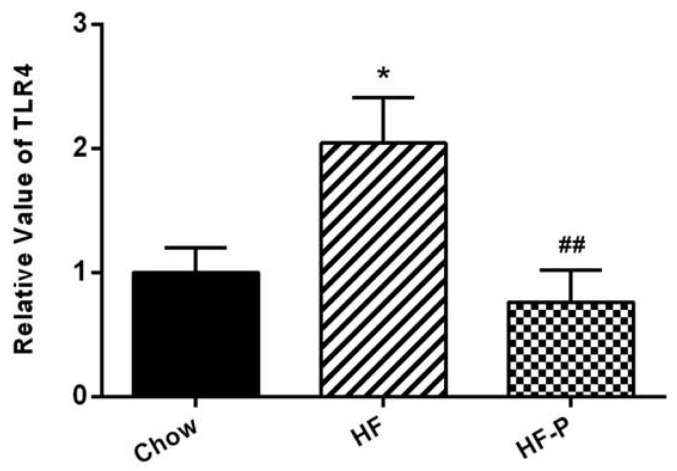
TLR4 mRNA expression in the ileal tissue of rats in the Chow, HF, and HF-P groups (* *p* < 0.05 *vs.* Chow, ## *p* < 0.01 *vs.* HF, one way ANOVA).

TLR4 mRNA levels in the ileal tissue of rats in the HF group were higher than those in the Chow group (2.04 ± 0.89 *vs.* 1.00 ± 0.49, *p* < 0.05). Pectin supplementation blunted this high fat diet induced increase of TLR4 mRNA in the HF-P group (0.76 ± 0.58 *vs.* 2.04 ± 0.89, *p* < 0.01) (Figure 5).

### 3.6. Apple-Derived Pectin Alleviated High Fat Diet Induced Ileal Inflammation in Rats

**Figure 6:**
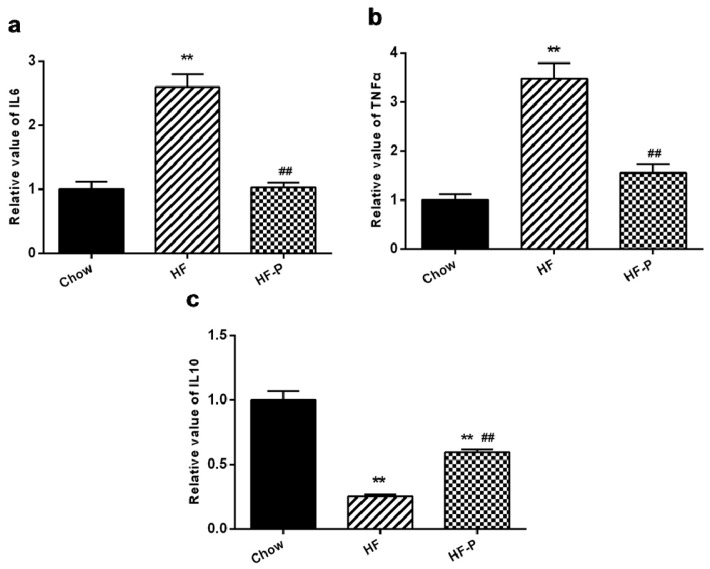
The mRNA levels of (**a**) tumor necrosis factor alpha (TNFα); (**b**) interleukin (IL)-6 and (**c**) IL-10 in the ileal tissue in Chow, HF, and HF-P groups (** *p* < 0.01 *vs.* Chow, ## *p* < 0.01 *vs.* HF, one way ANOVA).

The levels of pro-inflammation markers were significantly higher in the ileal issue of the HF group than in the Chow group (tumor necrosis factor alpha (TNFα), 3.48 ± 0.71 *vs.* 1.00 ± 0.27, *p* < 0.01; interleukin (IL)-6, 2.59 ± 0.45 *vs.* 1.00 ± 0.25, *p* < 0.01). The level of anti-inflammation cytokine IL-10 in the ileal issue was significantly lower in the HF group than that in the Chow group (IL-10, 0.25 ± 0.04 *vs.* 1.00 ± 0.20, *p* < 0.01) (Figure 6). The levels of TNFα and IL-6 were significantly decreased in the HF-P group as compared with the HF group (TNFα, 1.55 ± 0.37 *vs.* 3.48 ± 0.71, *p* < 0.01; IL-6, 1.02 ± 0.17 *vs.* 2.59 ± 0.45, *p* < 0.01), and the level of IL-10 in the ileal tissue was significantly increased in the HF-P group as compared with the HF group (0.60 ± 0.054 *vs.* 0.25 ± 0.04, *p* < 0.01) (Figure 6).

### 3.7. Apple-Derived Pectin Preserved Gut Barrier (Tight Junction) Function in Rats

**Figure 7:**
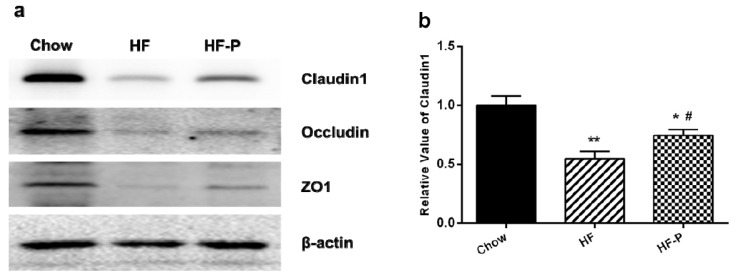

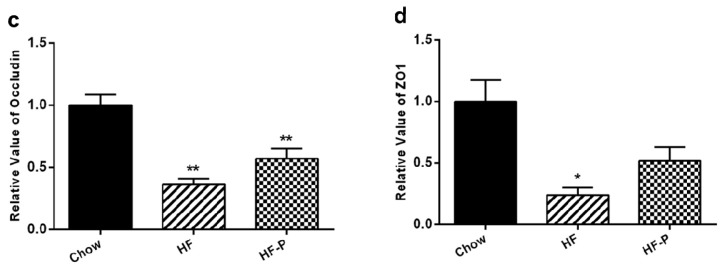
Expression levels of the tight junction proteins claudin1, occludin, and ZO-1. (**a**) Representative Western blots of claudin 1, occludin, and ZO1 with β-actin as a loading control; Quantitation of: (**b**) claudin 1; (**c**) occludin; and (**d**) ZO1 (** *p* < 0.01 *vs.* Chow, * *p* < 0.05 *vs.* Chow, # *p* < 0.05 *vs.* HF, one way ANOVA).

The expression levels of claudin1, occludin and zonula occludens-1 (ZO-1) proteins in rats of the HF group were significantly lower than those in the Chow group (claudin1, 0.55 ± 0.18 *vs.* 1.00 ± 0.22, *p* < 0.01; occludin, 0.36 ± 0.11 *vs.* 1.00 ± 0.23, *p* < 0.01; ZO-1, 0.24 ± 0.15 *vs.* 1.00 ± 0.40, *p* < 0.05). Supplementation of pectin significantly improved the level of claudin1 but only caused a tendency of increase in the levels of occludin and ZO-1 as compared with the high fat diet alone (claudin1, 0.76 ± 0.14 *vs.* 0.55 ± 0.18, *p* < 0.05; occludin, 0.57 ± 0.21 *vs.* 0.36 ± 0.11, *p* = 0.060; ZO-1, 0.52 ± 0.25 *vs.* 0.24 ± 0.15, *p* = 0.172) (Figure 7).

### 3.8. Apple-Derived Pectin Decreased High Fat Diet Induced Metabolic Endotoxemia

**Figure 8:**
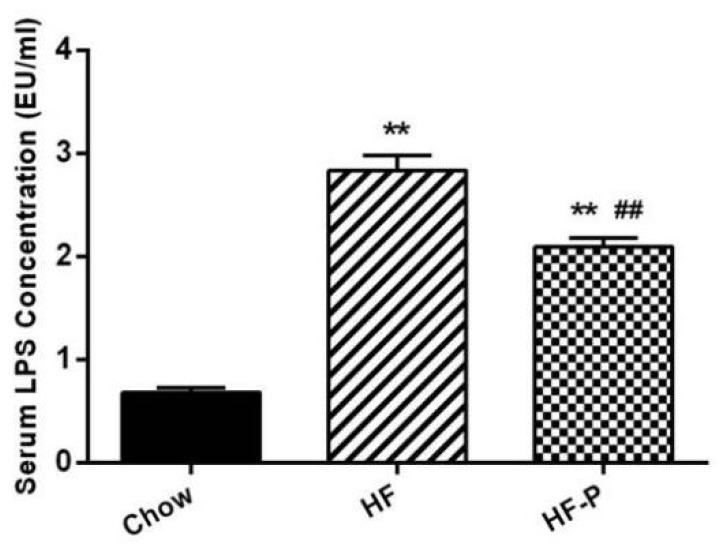
Serum LPS concentration (EU/mL) in Chow, HF, and HF-P groups. (** *p* < 0.01 *vs.* Chow, ## *p* < 0.01 *vs.* HF, one way ANOVA).

High fat diet caused a significant increase in the serum level of LPS as compared with chow diet (HF 2.83 ± 0.42 EU/mL *vs.* Chow 0.68 ± 0.14 EU/mL, *p* < 0.01) while supplementation of apple-derived pectin significantly decreased high fat diet induced LPS appearance in the serum (HF-P 2.09 ± 0.24 EU/mL *vs.* HF 2.83 ± 0.42 EU/mL, *p* < 0.01) (Figure 8).

### 3.9. Apple-Derived Pectin Alleviated High Fat Diet Induced Systemic Inflammation in Rats

**Figure 9:**
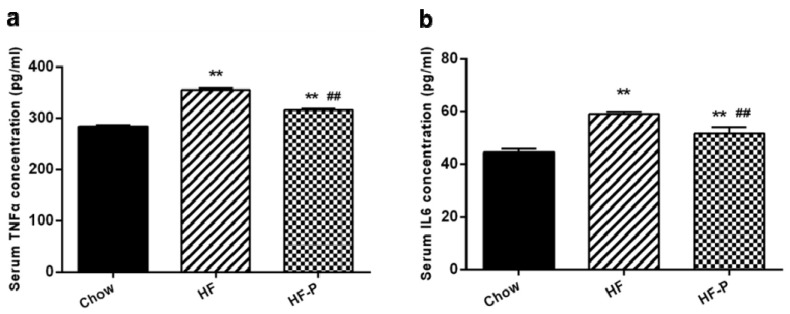
Portal serum levels of (**a**) TNFα and (**b**) IL-6 in Chow, HF, and HF-P groups (** *p* < 0.01 *vs.* Chow, ## *p* < 0.01 *vs.* HF, one way ANOVA).

The levels of pro-inflammation cytokines (TNFα and IL-6) in the portal serum of rats in the HF group were higher than those in the Chow group (TNFα: 355.59 ± 8.10 EU/mL *vs.* 283.16 ± 7.28 EU/mL, *p* < 0.01; IL-6: 58.98 ± 2.59 EU/mL *vs.* 44.56 ± 3.67 EU/mL, *p* < 0.01). Serum TNFα and IL-6 in the HF-P group were decreased compared with those in the HF group (TNFα: 316.13 ± 7.62 EU/mL *vs.* 355.59 ± 8.10 EU/mL, *p* < 0.01; IL-6: 51.78 ± 2.35 EU/mL *vs.* 58.98 ± 2.59 EU/mL, *p* < 0.01) (Figure 9).

## 4. Discussion

In this study, we found that rats fed with a high fat diet exhibited obvious increases of body weight and adipose tissue, disturbance of gut microbiota, gut barrier dysfunction, systemic chronic inflammation, and metabolic endotoxemia. However, these changes could be restored to normal levels by dietary supplementation with pectin. To the best of our knowledge, no previous study has investigated the potential effects of apple-derived pectin on obesity and how apple-derived pectin could modulate gut microbiota, gut barrier function, metabolic endotoxemia, and systemic inflammation in diet-induced obese rats.

Many studies have demonstrated that various types of dietary fiber play roles in anti-obesity and have hypoglycemic and hypolipidemic effects [31,32,33,34,35]. Similarly, we found in this study that supplementation with apple-derived pectin could significantly suppress weight gain and fat deposition in HFD fed rats. In addition, dyslipidemia, hyperglycemia, and hyperinsulinism caused by HFD were also alleviated with pectin supplementation to different extents. 

In 2004, Gordon *et al.* first reported that gut microbiota modulated lipid metabolism, suppressed activity of genes involved in fat consumption, and improved the activity of genes involved in fat synthesis, which led to excessive fat synthesis and fat accumulation in mice. They also found that the presence of gut microbiota was necessary for obesity occurrence, as germ free animals did not get obese even when fed a HFD [37]. Since then, additional studies have demonstrated that gut microbiota is an important factor when assessing risk factors associated with obesity and related disorders, such as dyslipidemia, hyperglycemia, inflammation and diabetes. Thus, modulation of gut microbiota might be a novel approach to manage body weight and metabolic disorders [35,38,39,40,41,42,43,44].

Here, we found that Bacteroidetes phylum, a principal component of gut microbiota, as well as subordinate Bacteroidia class, Bacteroidales order, Bacteroidaceae family, and *Bacteroides* genus, decreased sharply in rats fed with a high fat diet as compared to rats fed a normal diet. However, Firmicutes phylum, another principal component of gut microbiota, as well as subordinate Bacilli class, Lactobacillales order, *Lactococcus* genus and *Clostridium ruminantium* species, increased significantly in the HF group. Supplementation with apple-derived pectin in HFD fed rats restored bacteria levels to normal ranges (Chow group as reference).

Interestingly, Gammaproteobacteria and Deltaproteobacteria, two classes from the Proteobacteria phylum, presented different alteration trends, where the former increased and the latter decreased in the HF group as compared to the Chow group. As a result, the total level of Proteobacteria at the phylum level was similar among the three groups. Nevertheless, both Gammaproteobacteria and Deltaproteobacteria were restored to normal levels after supplementation with pectin.

Although not unanimously recognized, obesity is generally characterized by an increased ratio of Firmicutes to Bacteroidetes [45,46]. Similarly, we found here an increased level of Firmicutes and a lower level of Bacteroidetes. A previous study showed that some Lactobacillus species were associated with normal weight *(Bifidobacterium animalis* (*B. animalis*)) while others (*Lactobacillus reuteri* (*L. reuteri*)) were associated with obesity [47]. In this study, we observed changes in the level of *L. reuteri* in Bacilli class, Lactobacillales order and *Lactococcus* genus, which were restored with pectin supplementation. In addition, we report here for the first time changes in *Clostridium ruminantium* species in rats fed with HFD and attenuation with pectin supplementation. This finding needs to be investigated further for verification.

In previous studies, the expression of IAP and the activation of TLR4 were increased in the ileal tissue of obese rats, showing that activation of TLR4 could alter tight junctions and increase intestinal permeability [48]. IAP is a type of glycoprotein anchored in the apical membrane of intestine by a glycosyl-phosphatidyl-inositol linkage, which plays an important role in gut barrier function, including detoxification of bacterial LPS and free nucleotides [49,50], remission of intestinal inflammation [27,51], and modulation of gut microbiota [52,53,54,55]. Intercellular tight junctions play an important role in the permeability properties of the gut barrier [17,26]. Tight junctions consist of transmembrane proteins (occludin, claudins, and junctional adhesion molecule (JAM)), junctional complex proteins (such as ZO-1, zonula occludens-2 (ZO-2), Symplekin, and cingulin), and actin cytoskeleton [56]. Transmembrane proteins act as a mediator in adhesion, gut barrier formation, selective paracellular diffusion, interaction with junctional complex proteins, and actin cytoskeleton, which is significant for the regulation of the permeability of gut barrier [26].

In this study, we observed that increased expression of TLR4 mRNA in the ileum tissue of HFD fed rats was blunted upon pectin supplementation (Figure 5). We also observed that expression of IAP as well as the tight junction proteins claudin 1, occludin, and ZO-1 were significantly reduced in comparison with the chow group (Figure 4). Notably, supplementation with pectin restored claudin 1 and IAP to normal levels, and caused a tendency of increase in the levels of occludin and ZO-1. One possible explanation for this ineffectiveness of pectin to restore occludin and ZO-1 is that the duration of HFD was too long so that the damage was irreversible. Taken together, our data suggest that apple-derived pectin improves gut barrier function and maintains the integrity of intestine. 

In previous studies, inulin-type fructan and wheat-derived arabinoxylan oligosaccharides were shown to modulate gut microbiota in cecal content and increase integrated gut barrier function, leading to improvements in metabolic endotoxemia and inflammation [9,35,57]. In the present study, we observed that the level of endotoxin in the portal serum was significantly reduced with pectin supplementation (Figure 3). In addition, we observed that two pro-inflammatory factors, namely TNFα and IL-6, were downregulated in the portal serum upon pectin supplementation. The HFD-induced increase in TNFα and IL-6 mRNA in the ileal tissue was also blunted with pectin. Meanwhile, pectin upregulated the mRNA expression of the anti-inflammatory factor IL-10 in the ileal tissue of obese rats, increasing the anti-inflammatory effect. In accordance with a decrease in metabolic endotoxemia, we propose that the lower inflammatory tone observed upon pectin supplementation was due to modulation of inflammatory factors. 

It is well known that inflammatory cytokines TNFα and IL-6 could cause insulin resistance [58,59,60]. Consist with the effects on TNFα and IL-6, pectin supplementation could mildly alleviate insulin resistance in HFD fed rats as evidenced by changes in serum total cholesterol, triglycerides, glucose and insulin (*p* < 0.01 for total cholesterol, however, only a trend for triglycerides, glucose, and insulin). These findings are consistent with other studies that showed consumption of different kinds of dietary fiber, such as fractionated yellow pea fiber, oligofructose, and grape skin extract could lower blood glucose levels in human subjects and rodents [33,34,61,62,63].

It is known that insulin functions to promote fat synthesis, transfer glucose into cells, and promote glycogen synthesis. In a state of hyperinsulinism and glycemia, more fat deposition occurs, leading to obesity. In accordance with our hypothesis, the increases in body weight and fat pad weight in the HF group were blunted with pectin supplementation.

**Figure 10:**
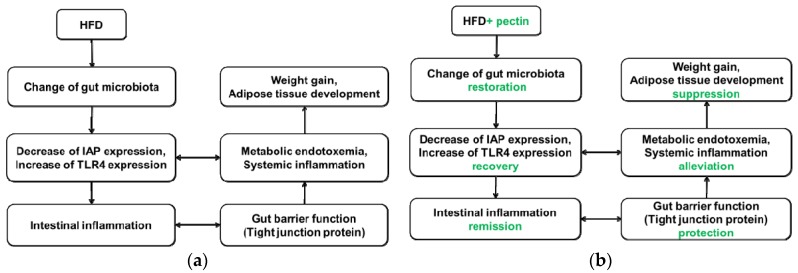
(**a**) Proposed model by which HFD leads to weight gain and adipose development. HFD leads to disturbance of gut microbiota, possibly by decreasing IAP expression and increasing TLR4 expression, which may result in metabolic endotoxemia and intestinal and systemic inflammation. Intestinal inflammation results in altered gut barrier function and promotes penetration of LPS from the lumen to the lamina propria. The precise mechanism by which metabolic endotoxemia leads to weight gain and adipose tissue development is not clear; (**b**) Proposed model by which supplementation of apple-derived pectin suppresses weight gain and adipose development. Pectin supplementation maintains gut microbiota, promoting recovery of IAP and TLR4 levels, which may alleviate metabolic endotoxemia and intestinal and systemic inflammation. Thus, gut barrier function is protected and penetration of LPS from the lumen to the lamina propria is suppressed. These events lead to suppression of weight gain and adipose development.

This study demonstrated a complex link between obesity and gut microbiota, gut barrier, inflammation, and metabolic endotoxemia. The sequence of events could possibly occur as follows: modulation of gut microbiota, expression of IAP and TLR4, intestinal inflammation, altered gut barrier function (especially tight junction), change in serum LPS concentration, and, finally, increases in weight gain and development of adipose tissue (see Figure 10).

However, there are some limitations associated with our study, which warrants precautions in interpretation of the data. For instance, gut microbiota are different in human and rats, and our conclusion in rats may not be readily translatable into human. Given our positive results in rats, it is worthwhile to carry out clinical trials to properly address whether apple-derived pectin also has such beneficial effects in human. Moreover, since we did not transplant the possible “obesity-causing” microbiota to sterile rats for further verification, we could not establish causality between gut microbiota and obesity development. We also realize the potential limitation of our method for measurement of serum LPS. This method determines the bio-reactivity of LPS rather than its absolute quantity. Although the bio-reactivity of LPS in different groups of rats correlated with the changes in gut barrier function, we cannot rule out the possibility that changes in gut microbiota might directly cause the differences in the bio-reactivity of LPS in serum. Nevertheless, apple-derived pectin could alleviate HFD-induced metabolic endotoxemia.

## 5. Conclusions

In conclusion, this study demonstrated that apple-derived pectin could modulate gut microbiota, as previously shown for inulin-type fructan and wheat-derived arabinoxylan oligosaccharides [35,64]. Concomitantly, pectin supplementation alleviated HFD-induced body weight gain, fat mass development, dyslipidemia, hyperglycemia, hyperinsulinism, metabolic endotoxemia, and systemic inflammation in obese rats. In addition, expression of IAP and gut barrier function (tight junctions) were improved with pectin supplementation. These results indicate that apple-derived pectin might play a protective role with prebiotic properties in the prevention of obesity and associated metabolic and inflammatory disorders. Prospectively, it might become a useful tool for clinical management of patients with metabolic disorders.

## Figures and Tables

**Table 1:** Primer for quantitative real-time polymerase chain reaction.

Primer	Sequence
TNFa Forward	AAATGGGCTCCCTCTCATCAGTTC
TNFa Reverse	TCTGCTTGGTGGTTTGCTACGAC
IL6 Forward	AGCCAGAGTCATTCAGAGCA
IL6 Reverse	AGAGCATTGGAAGTTGGGGT
IL10 Forward	GTTGCCAAGCCTTGTCAGAA
IL10 Reverse	GGGAGAAATCGATGACAGCG
TLR4 Forward	TTCCTTTCCTGCCTGAGACC
TLR4 Reverse	CATGCCATGCCTTGTCTTCA
βactin Forward	GAGAGGGAAATCGTGCGTGACA
βactin Reverse	GTTTCATGGATGCCACAGGAT
36B4 Forward	TAAAGACTGGAGACAAGGTG
36B4 Reverse	GTGTACTCAGTCTCCACAGA

## Supplementary Materials

The following are available online at http://www.mdpi.com/2072-6643/8/3/126/s1, Table S1: Total pairs read number and raw data per sample.

## Abbreviations

The following abbreviations are used in this manuscript:

ANOVA	Analysis of variance
CHD	Coronary heart disease
HFD	High-fat diet
HF-P	High-fat diet supplemented with pectin
IAP	Intestinal alkaline phosphatase
IL	Interleukin
LPS	Lipopolysaccharide
T2DM	Type 2 diabetes mellitus
TLR4	Toll-like receptor 4
TNFα	Tumor necrosis factor alpha
