# Supplementary material for: Apple-Derived Pectin Modulates Gut Microbiota, Improves Gut Barrier Function, and Attenuates Metabolic Endotoxemia in Rats with Diet-Induced Obesity
Source: Nutrients. 2016 Feb 29;8(3):126. doi: 10.3390/nu8030126 (PMC4808856; doi:10.3390/nu8030126)
Supplement: Supplementary file 1 [file nutrients-08-00126-s001.docx]

Supplementary Materials: Apple-Derived Pectin Modulates Gut Microbiota, Improves Gut Barrier Function, and Attenuates Metabolic Endotoxemia in Rats with Diet-Induced Obesity

Tingting Jiang, Xuejin Gao, Chao Wu, Feng Tian, Qiucheng Lei, Jingcheng Bi, Bingxian Xie, Hongyu Wang, Shuai Chen and Xinying Wang

**Table S1.** Total pairs read number and raw data per sample.

| **Sample Name** | **Total Pairs Read Number** | **Raw Data (Mbp)** |
| --- | --- | --- |
| Chow1 | 32,491 | 19.39 |
| Chow2 | 32,237 | 18.87 |
| Chow3 | 32,459 | 18.23 |
| Chow4 | 31,952 | 18.24 |
| Chow5 | 31,914 | 18.62 |
| Chow6 | 32,604 | 21.00 |
| HF1 | 32,494 | 17.13 |
| HF2 | 32,134 | 17.09 |
| HF3 | 32,295 | 17.17 |
| HF4 | 32,127 | 17.55 |
| HF5 | 32,465 | 17.21 |
| HF6 | 32,577 | 17.60 |
| HF7 | 32,129 | 17.49 |
| HF8 | 32,274 | 17.19 |
| HF-P1 | 32,173 | 17.70 |
| HF-P2 | 32,016 | 19.91 |
| HF-P3 | 32,386 | 17.57 |
| HF-P4 | 32,466 | 18.40 |
| HF-P5 | 32,421 | 18.21 |
| HF-P6 | 32,322 | 18.18 |
